# A traditional Chinese medicine therapy for coronary heart disease after percutaneous coronary intervention: a meta-analysis of randomized, double-blind, placebo-controlled trials

**DOI:** 10.1042/BSR20180973

**Published:** 2018-10-15

**Authors:** Ruixue Chen, Ya Xiao, Minghao Chen, Jingyi He, Mengtian Huang, Xitao Hong, Xin Liu, Taoran Fu, Jingzhi Zhang, Liguo Chen

**Affiliations:** 1 Department of Traditional Chinese Medicine, The Second Affiliated Hospital, Guangzhou Medical University, Guangzhou, Guangdong 510260, China; 2 School of Chinese Medicine, Jinan University, Guangzhou, Guangdong 510632, China; 3 Reproductive Center, Guangdong Women and Children Hospital, Guangzhou, Guangdong 511400, China; 4 School of Basic Medicine, Jinan University, Guangzhou, Guangdong 510632, China; 5 Guangzhou Institute of Cardiovascular Disease, The Second Affiliated Hospital, Guangzhou Medical University, Guangzhou, Guangdong 510260, China

**Keywords:** Coronary heart disease, Huoxue Huayu therapy, Meta-analysis, Percutaneous coronary intervention

## Abstract

Huoxue Huayu therapy (HXHY) has been widely used to treat cardiovascular diseases in traditional Chinese medicine (TCM) such as hypertension and coronary heart disease (CHD). The present study describes a meta-analysis of a series of prospective randomized, double-blind, placebo-controlled trials conducted to evaluate the effect of HXHY on patients with CHD after percutaneous coronary intervention (PCI). The Cochrane Library, PubMed, EMBASE, the China National Knowledge Infrastructure (CNKI), the Chinese Biomedical Literature database, and the Wanfang database were searched up until June 2018. A series of randomized controlled clinical trials were included and the subjects were patients with CHD who had undergone PCI. The experimental group was treated with HXHY therapy, and the control group was treated with placebo; meanwhile, all the patients accepted conventional Western medicine. Review Manager 5.3 software was used for the statistical analysis. Ten trials were included in the final study. The overall risk of bias assessment was low. HXHY had a greater beneficial effect on reducing the in-stent restenosis (ISR) rate (RR = 0.57, 95% confidence interval [CI] [0.40–0.80], *P*=0.001) and the degree of restenosis (MD = −8.89, 95% CI [−10.62 to −7.17], *P*<0.00001) compared with Placebo. Moreover, HXHY was determined to be more effective in improving Seattle Angina Questionnaires (SAQ) and the revascularization rate (RR = 0.54, 95% CI [0.32–0.90], *P*=0.02) compared with Placebo, whereas the rate of death and MI of patients treated with HXHY were no different from those treated with the placebo (*P*>0.05). Therefore, HXHY is an effective and safe therapy for CHD patients after PCI.

## Introduction

Coronary heart disease (CHD) is one of the most serious cardiovascular diseases (CVDs) threatening human health [1]. The World Health Organization (WHO) estimates that approximately 17 million people die of CVDs in the world every year and 7.4 million die of CHD. It is estimated that almost 23.6 million people will die from CVDs by 2030 [2]. In 2016, the American Heart Association (AHA) showed that there were 400,000 deaths due to CHD each year in the United States, and the number of new cases of CHD is up to 785,000 [3]. The number of deaths from cardiovascular disease accounted for 34% of the total number of deaths in the United Kingdom and 35% in Australia [4]. The cost of treating CHD is quite expensive and it has created a heavy burden for patients and society.

Presently, percutaneous coronary intervention (PCI) is the most common method for treating CHD, which could significantly reduce the mortality of CHD. After bare metal stent implantation, the in-stent restenosis (ISR) rate was 20–30%, and the ISR rate of a drug eluting stent was reduced to approximately 10% [5,6]. ISR increased the rate of major adverse cardiovascular events (MACE) and influenced the quality of patients’ life [7]. The European Society of Cardiology (ESC) recommended that patients with CHD take dual antiplatelet therapy (DAPT) after PCI. That is, patients need to take clopidogrel and aspirin for 9–12 months to reduce the rate of MACE [8]. The United States Food and Drug Administration (FDA) noted that prolonging the application of DAPT has potential benefits on patients, whereas studies showed that the prolonged application of the maintenance dose could cause leukopenia, and there were still 10–15% of patients who experienced myocardial infarction (MI), stroke, and death [9–11].

CHD is a chronic disease and the cost is expensive, the alternative and complementary treatments play more and more important roles in treating CHD. Based on the basic theories of traditional Chinese medicine (TCM), CHD is equivalent to the term of ‘Xiong Bi’, which was described in the ‘Inner Canon of Yellow Emperor’ (Chinese name in pinyin is ‘Huang Di Nei Jing’) in the Western Han Dynasty and is particularly discussed in ‘Synopsis of Golden Chamber’ (Chinese name in pinyin is ‘Jin Gui Yao Lüe’) in the Eastern Han Dynasty. The etiology and pathogenesis of CHD are related to blood stasis, which blocks the heart and vessels. Therefore, activating circulation and removing blood stasis (Chinese name in pinyin is ‘Huoxue Huayu’) is an important therapy for CHD. In the Qing Dynasty, the Xuefu Zhuyu Decoction was used to treat CHD and originated from the book ‘Correction on Errors in Medical Classics’ (Chinese name in pinyin is ‘Yi Lin Gai Cuo’), which has been used for hundreds of years. Although PCI could dredge the narrow obstruent heart and vessels and improve the symptoms of angina pectoris, it cannot change the etiology of CHD. Huoxue Huayu therapy (HXHY) is widely used to treat patients with CHD after PCI. Clinical studies have proven that HXHY could effectively prevent and treat ISR with no-reflow and myocardial injury after PCI [12,13]. However, the current state of evidence of HXHY for CHD has been so far unknown. Therefore, we conducted a meta-analysis of randomized, double-blind, placebo-controlled trials to evaluate the efficacy and safety of HXHY on patients with CHD after PCI.

## Methods

### Search strategy

The Cochrane Library (1993–2018), PubMed (1989–2018), EMBASE (1947–2018), the China National Knowledge Infrastructure database (1979–2018), the Chinese Biomedical Literature database (1990–2018), the VIP database (1989–2018), and the Wanfang database (1982–2018) were searched. The search terms used were (Chinese medicine OR Chinese patent medicine OR herbs OR herbal formula OR Huoxue OR Huayu) AND (CHD OR coronary heart disease OR CAD OR coronary artery disease) AND (PCI OR percutaneous coronary intervention OR intervention therapy OR interventional treatment) AND (randomized controlled trial AND (double-blind trial OR placebo trial)). No limit was placed on the language.

### Study selection

Studies were selected according to the Cochrane Handbook for Systematic Reviews of Interventions [14]. Studies meeting the following criteria were included: (i) the studies were performed as randomized, double-blind, or placebo controlled trials; (ii) patients were diagnosed with CHD and received PCI; (iii) DAPT (clopidogrel and aspirin) plus other Western medicine was permitted to be taken according to individual symptoms; and (iv) HXHY therapy was used for the experimental group and a placebo for the control group. The ISR was recorded as the primary outcome. The Seattle Angina Questionnaires (SAQ) [15] and MACE improvement were recorded as secondary outcome measures.

### Data abstraction

Three authors independently screened the literature titles and abstracts and then reviewed the full text and evaluated the studies that met the inclusion criteria. Then data, such as study design, randomization, sample size, treatment, HXHY formula, dosage forms, ingredients of the formula, treatment duration and outcomes, were extracted from the included studies. Data were extracted as intention-to-treat analyses (ITT) in which dropouts were assumed to be treatment failures if trial reporting allowed this technique. Disagreements were resolved by RXC. Assessment of the methodological quality was conducted by RXC, YX, and MHC according to the Cochrane Collaboration tool.

### Data synthesis and analysis

Relative risk (RR), weighted mean difference (MD), and 95% confidence intervals (CI) were reported for the outcomes. Heterogeneity of the included studies was conducted by a chi-square test and the inconsistency index statistic (*I*^2^). If no heterogeneity occurred (*I*^2^ < 50% or *P*>0.05), a fixed-effect model was used to calculate the pooled RR and MD. If substantial heterogeneity occurred (*I*^2^ > 50% or *P*<0.05), a random-effect model was used to calculate the pooled RR and MD. Review Manager 5.3 was used for analyses, and the results were presented by forest plot.

## Results

### Included studies

**Figure 1:**
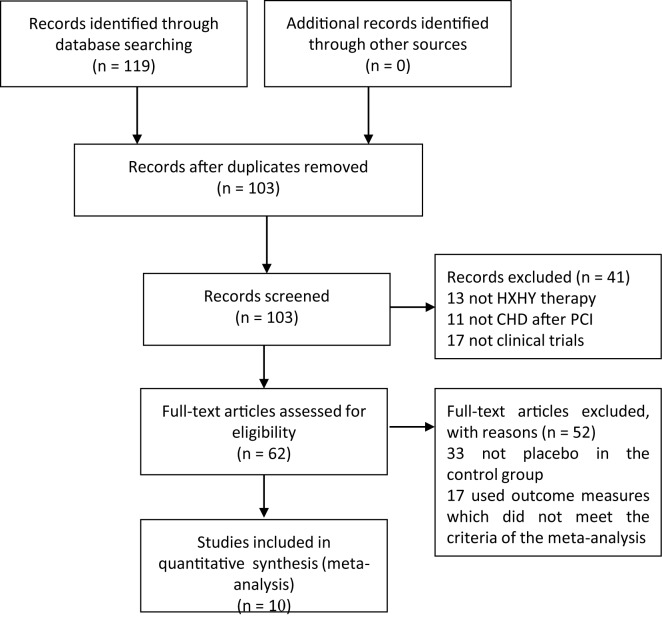
Flow chart of the study selection

A total of 119 studies were identified and screened by computer search. A total of 16 articles were duplicates, and 41 articles were excluded after reviewing the abstracts. After reviewing, ten studies were included for the meta-analysis (Figure 1); these ten studies included 920 patients in the experimental group and 886 patients in the control group. Characteristics of the ten included studies are shown in Table 1 and the ingredients of the HXHY formula are listed in Table 2.

**Table 1.** Characteristics of the included studies

Study	Population	Age (years)	Man (%)	*N* (HXHY compared with Placebo)	Classification of CHD (%)	HXHY (form)	Doses	Duration
**Chen, 2005 [16]**	Multi-centre	58.63	78.66	154 compared with 154	AP (60.83) MI (39.17)	Xiong Shao capsules	2 capsules, t.i.d.	24 weeks
**Qiao, 2005 [17]**	Single centre	64.84	59.32	30 compared with 29	AP (64.41), MI (35.59)	Tong Guan capsules	3 capsules, t.i.d.	4 weeks
**Chu, 2010 [18]**	Single centre	60.22	63.33	28 compared with 29	AP (100)	Xuefu Zhuyu capsules	3 capsules, t.i.d.	4 weeks
**Zhang, 2011 [19]**	Multi-centre	58.04	85.84	108 compared with111	MI (100)	Tong Xin Luo (capsules)	4 capsules, t.i.d.	24 weeks
**Chen, 2013 [20]**	Single centre	64.65	55.0	30 compared with 30	AP (50), MI (50)	Tong Guan capsules	3 capsules, t.i.d.	12 weeks
**Wu, 2014 [21]**	Multi-centre	60.29	77.97	294 compared with 295	AP (56.41), MI (43.59)	Huxin formula (granules)	10 g, s.i.d.	24 weeks
**Lu, 2014 [22]**	Single centre	61.0	52.22	90 compared with 90	AP (54.44), MI (27.22)	Tong Xin Luo (capsules)	3 capsules, t.i.d.	52 weeks
**Xu, 2015 [23]**	Multi-centre	69.45	73.26	113 compared with 74	AP (100)	Shenzhu Guanxin recipe (granules)	12 g, s.i.d.	12 weeks
**Ma, 2015 [24]**	Multi-centre	57.59	76.81	34 compared with 35	AP (79.71), MI (20.29)	Guan Jie Ling (decoction)	2.5 ml/kg, t.i.d.	8 weeks
**Qi, 2016 [25]**	Single centre	58.35	57.5	39 compared with 39	AP (100)	Buyang Huanwu decoction	1 package, b.i.d.	2 weeks

Abbreviations: AP, angina pectoris; MI, myocardial infarction.

**Table 2.** The ingredients of each HXHY formula

Formula	Ingredients of each HXHY formula (Chinese name in ‘pinyin’)			
**Xiong Shao capsules**	*Ligusticumwallichii* (Chuan Xiong)	*Radix paeoniaerubrathe* (Chi Shao)		
**Tong Guan capsules**	*Salvia miltiorrhiza* (Dan Shen)	*Radix astragali* (Bei Qi)	*Hirudenipponica* (Shui Zhi)	*Dryobalanopsaromatica* (Bing Pian)
**Xuefu Zhuyu capsules**	*Prunus persica* (Tao Ren)	*Carthamustinctorius* (Hong Hua)	*Radix paeoniaerubrathe* (Chi Shao)	*Angelica sinensis* (Dang Gui)
	*Ligusticumwallichii* (Chuan Xiong)	*Radix bupleuri* (Chai Hu)	*Platycodon grandiflorum* (JieGeng)	*Fructusaurantii* (Zhi Qiao)
	*Rehmanniaglutinosa* (Sheng Di Huang)	*Radix achyranthisbidentatae* (Niu Xi)	*Radix liquiritiae* (Gan Cao)	
**Tong Xin Luo**	*Radix paeoniaerubrathe* (Chi Shao)	*Panax ginseng* (Ren Shen)	*Hirudenipponica* (Shui Zhi)	*Buthusmartensii* (Quan Xie)
	*Eupolyphagasinensis* (Tu Bie Chong)	*Scolopendrasubspinipesmutilans* (Wu Gong)	*Cryptotympanapustulata* (Chan Tui)	*Dryobalanopsaromatica* (Bing Pian)
**Huxin formula**	*Panax ginseng* (Ren Shen)	*Exocarpiumcitri* (Ju Hong)	*Panaxnotoginseng*(San Qi)	*Salvia miltiorrhiza* (Dan Shen)
	*Pinellia ternate* (Ban Xia)	*Pogostemoncablin* (Huo Xiang)		
**Shenzhu Guanxin recipe**	*Panax ginseng* (Ren Shen)	*Panaxnotoginseng* (San Qi)	*Rhizomaatractylodis* (Cang Zhu)	*Hirudenipponica* (Shui Zhi)
	*Pinellia ternate* (Ban Xia)	*Panax quinquefolium* (Xi Yang Shen)	*Folium nelumbinis* (He Ye)	
**Guan Jie Ling**	*Astragalus memeranaceus* (Huang Qi)	*Panax ginseng* (Ren Shen)	*Prunus persica* (Tao Ren)	*Carthamustinctorius* (Hong Hua)
	*Angelica sinensis* (Dang Gui Wei)	*Ligusticumwallichii*(Chuan Xiong)	*Buthusmartensii* (Quan Xie)	*Scolopendrasubspinipesmutilans* (Wu Gong)
	*Gekkoswinhonis* (Bi Hu)	*CollaCornus Cervi* (Lu Jiao Jiao)	*Pinellia ternate* (Ban Xia)	*Scutellariabaicalensis* (Huang Qin)
	*Cinnamomum cassia* (Gui Zhi)	*Radix liquiritiae* (Gan Cao)	*Rhizomacoptidis* (Huang Lian)	*Rhizomazingiberis* (Gan Jiang)
	*FructusZiziphiJujubae* (Da Zao)	*Rehmanniaglutinosa* (Sheng Di Huang)	*Radix paeoniaerubrathe* (Chi Shao)	
**Buyang Huanwu decoction**	*Astragalus memeranaceus* (Huang Qi)	*Radix paeoniaerubrathe* (Chi Shao)	*Ligusticumwallichii*(Chuan Xiong)	*Angelica sinensis* (Dang Gui Wei)
	*Prunus persica* (Tao Ren)	*Carthamustinctorius* (Hong Hua)	*Pheretima aspergillum* (Di Long)	*Lignum acronychiae* (Jiang Xiang)
	*Corydalis yanhusuo* (Yan Hu Suo)			

### Risk of bias

**Figure 2:**
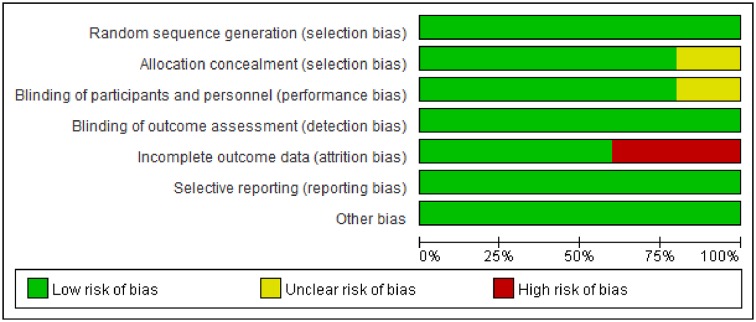
Risk of bias graph

The results of the bias risk assessment showed a high quality for the studies (Figure 2). A low risk of bias was found across the studies for random sequence generation, blinding of outcome assessment, and selective reporting. There were no details about allocation concealment in Chen et al. [20] and Lu et al. [22], and no details about placebo in Chen et al. [16], Zhang [19], and Qi and Song [25]. In Chen et al. [16], Wu et al. [21], Ma and Yuan [24] and Qi and Song [25], attrition bias was rated as high risk ascribing to a lack of ITT analysis. Furthermore, it is showed that all the included studies were supported by various foundation, except for Lu and Ma. The funding of the included studies is as follows: Chen was funded by the National Medical Science and Technique Foundation during the ‘9th Five-Year-Plan’; Qiao was funded by the Social Development program of Department of Science and Technology of Guangdong Province; Chu and Chen were funded by the National Natural Science Foundation of China; Zhang was funded by National Program on Key Basic Research Project of China; Wu was funded by National Key Technology Research and Development Program of China during ‘10th Five-Year-Plan’; Xu was funded by the Science and Technology program of Guangdong Province; Qi was funded by the Special Research Project of the construction of National Traditional Chinese Medicine Clinical Research Base; and there were no details about funds in Lu and Ma.

### In-stent restenosis

**Figure 3:**
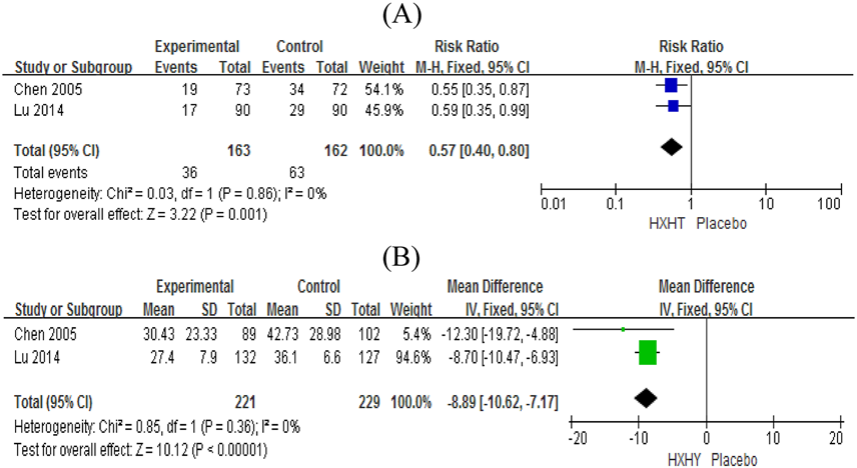
Forest plot of primary outcomes. (**A**) Forest plot of the ISR rate with weights from fixed effects analysis. (**B**) Forest plot of the degree of restenosis with weights from fixed effects analysis.

Two studies reported the ISR rate and the degree of restenosis [16,22]. Compared with the placebo, patients treated with HXHY showed a significant improvement in the ISR rate (RR = 0.57, 95% CI [0.40–0.80], *P*=0.001), and no substantial heterogeneity was found (*P*=0.86, *I*^2^ = 0%); patients treated with HXHY showed a significant improvement in the degree of restenosis (MD = −8.89, 95% CI [−10.62 to −7.17], *P*<0.00001), and no substantial heterogeneity among the studies was found (*P*=0.36, *I*^2^ = 0%) (Figure 3).

### Seattle Angina Questionnaires

Four studies were evaluated for SAQ improvement [17,18,23,25], including physical limitation (PL), angina stability (AS), angina frequency (AF), treatment satisfaction (TS), and disease perception (DP). Compared with the placebo, patients treated with HXHY showed a significant improvement in AS (MD = 15.87, 95% CI [5.20–26.55], *P*=0.004), TS (MD = 11.69, 95% CI [4.37–19.01], *P*=0.002), and DP (MD = 4.00, 95% CI [0.80–7.20], *P*=0.01). There was significant heterogeneity across the studies (Table 3).

**Table 3.** SAQ results: HXHY compared with Placebo

SAQ	Heterogeneity		Effect value	
	*I*^2^	*P*	MD [95% CI]	*P*
PL	86%	0.0000*	2.41 [−5.27 to 10.09]	0.54
AS	90%	0.0000*	15.87 [5.20 to 26.55]	0.004*
AF	83%	0.0005*	6.24 [−4.59 to 17.08]	0.26
TS	86%	0.0000*	11.69 [4.37 to 19.01]	0.002*
DP	41%	0.17	4.00 [0.80 to 7.20]	0.01*

*P<0.05, there was a statistical significance.

### Major adverse cardiovascular events

**Figure 4:**
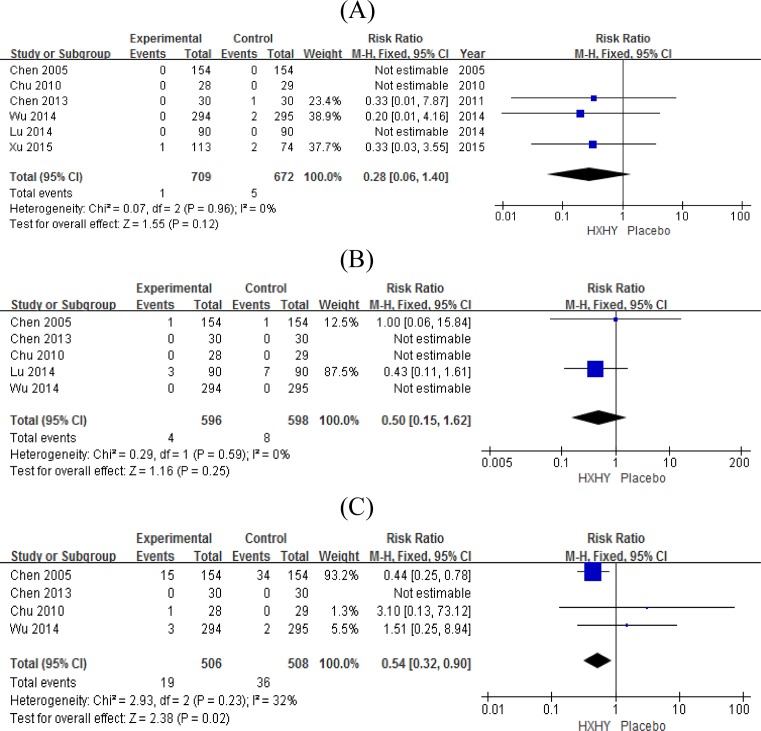
Forest plot of secondary outcomes. (**A**) Forest plot of the death rate with weights from fixed effects analysis. (**B**) Forest plot of the non-fatal MI rate with weights from fixed effects analysis. (**C**) Forest plot of the revascularization rate with weights from fixed effects analysis.

Six studies reported MACE [16,18,20,21–23], including death, non-fatal MI, and revascularization. Compared with the placebo, patients treated with HXHY showed no significant improvement in the death rate (RR = 0.28, 95% CI [0.06–1.40], *P*=0.12) or non-fatal MI rate (RR = 0.50, 95% CI [0.15–1.62], *P*=0.25); however, patients treated with HXHY showed a significant improvement in the revascularization rate (RR = 0.54, 95% CI [0.32–0.90], *P*=0.02). There was no significant heterogeneity across the studies (Figure 4).

### Safety profile

Four studies reported the occurrence of adverse events [16,18,19,24]. Chen 2005 reported that 2 out of 308 participants had stomach discomfort, but the symptoms soon disappeared; 1 out of 154 participants was diagnosed with granulocytopenia in the control group, but it might have been related to taking Western medicine [16]. Chu et al. reported 1 participant who had stomach discomfort and dropped out [18]. Zhang reported that 10 out of 219 participants experienced a variety of adverse events, including nausea, stomachache, cardiac failure, cerebral hemorrhage, and death, whereas the number of adverse events reported by patients treated with HXHY was not significantly different from those reported by patients treated with the placebo [19]. Ma and Yuan reported that 1 participant developed a rash and 1 developed mild abdominal pain in the HXHY group; 2 participants developed a rash, and seven developed gastrointestinal reactions in the placebo group [24].

## Conclusions and discussion

According to the ten randomized, double-blind, placebo-controlled trials, patients treated with HXHY showed a significant improvement in ISR rate (RR = 0.57) and the degree of restenosis (MD = −8.89), quality of life estimated by SAQ and the revascularization rate (RR = 0.54) compared with the placebo. No evidence of publication bias in this analysis was found. The number of reported adverse events associated with HXHY was similar to that associated with the placebo. Therefore, it suggests that HXHY is a safe and effective therapy for treating patients with CHD after PCI.

CHD develops from coronary thrombosis and inflammatory reactions, which result from a variety of bioactive substances released by abnormally activated platelets. Therefore, the pathological basis of coronary thrombosis is abnormal platelet activation and an inflammatory reaction. As the main treatment for CHD, PCI did not change the pathological state, therefore, the stent thrombosis and ISR might be recurrent and lead to adverse cardiovascular events. Therefore, antiplatelet drugs after PCI are needed. DAPT, aspirin, and clopidogrel are recommended for treating CHD after PCI. Clopidogrel is a receptor antagonist of adenosine diphosphate (ADP) and could inhibit platelet aggregation induced by ADP and thus inhibit the development of coronary thrombosis. Aspirin couldn’t inhibit platelet aggregation induced by ADP, but the combination of aspirin and clopidogrel could inhibit thrombin and platelet activation. However, clopidogrel resistance often occurs in clinical applications. Then, patients need to increase the dosage of clopidogrel, apply triple antiplatelet drugs (aspirin, clopidogrel, and cilostazol), take a receptor antagonist of P2Y12, or seek help from TCM [26–29]. For example, China FDA recommended more than 200 Chinese patent medicines as complementary or adjunctive therapies for angina pectoris in mainland China, which play an active role in reducing the incidence of primary endpoint events, decreasing anginal attack rate, and improving electrocardiogram [30].

In TCM, blood stasis, which blocks the heart and vessels, is the main pathogenesis of CHD after PCI. Modern pharmacological studies proved that HXHY could effectively improve the etiology and pathogenesis of CHD, which was beneficial to long-term efficacy. *Tongguan Capsule* could improve the postoperative hypercoagulable state, regulate the coagulation-fibrinolysis system, and inhibit the thrombus formation by inhibiting platelet activation [31,32]. *Xuefu Zhuyu capsule* could inhibit inflammatory reactions by decreasing the expression of inflammatory factors, such as high-sensitivity C-reactive protein (Hs-CRP), interleukin-18 (IL-18), and plague destabilizing factors, including homocysteine (Hcy) and metal matrix proteinase-9 (MMP-9) [18]. *Guan Jie Ling* could inhibit inflammatory reactions by decreasing the expression of proinflammatory and procoagulant cytokines, including tumor necrosis factor (TNF-α), interleukin-6 (IL-6), and tissue factor (TF), and increasing the expression of anti-inflammatory cytokines and tissue factor pathway inhibitor (TFPI) [24]. It is proved that the ameliorative effects of *Buyang Huanwu Decoction* on CHD rats with BSS are mediated by the improvement of hemorheological disorders and energy metabolism [33]. These experiments proved that HXHY was significant in treating CHD, ISR, and promoting angiogenesis.

There was significant heterogeneity for the SAQ. We checked the selected and included studies and found that the Chinese herb medicine were different among the four studies [17,18,23,25]. This may contribute to the heterogeneity. Moreover, the treatment time of HXHY in each study was different; in the Qiao, Chu, and Xu studies, the treatment time was 1 month, whereas in the Qi study, it was 2 weeks. Thus, the treatment time of HXHY may be a source of heterogeneity. Although the methodological quality of the included studies was high, there was still a potential bias. Four trials were based on per-protocol analysis instead of ITT analysis [16,21,24,25]. This might lead to an increase in the possibility of false-positive results. Another flaw in the quality of the included studies was unclear details of the placebo, including its chemical properties, appearance, and taste [16,19,25]. This might cause unblinding interference to investigators or patients and influence the final outcomes.

There are some limitations in the meta-analysis. First, the number of included trials and the sample size were small. It would be necessary to evaluate the effect size of HXHY when large sample trials are conducted in the future. Due to the small number of included trials and sample size, there might be publication bias in the meta-analysis. Second, there were eight HXHY formulas in all the included trials in which the formula compositions, doses, and dosage forms were different, which might lead to a heterogeneous result.

In the meta-analysis, DAPT plus HXHY can significantly improve the ISR, quality of life and revascularization of patients with CHD after PCI. However, due to the limitation of TCM, reports of HXHY for CHD patients are mostly in Chinese. Therefore, further standardized, randomized, double-blind, multi-center trials are required to popularize the clinical application of HXHY for patients with CHD after PCI.

## Abbreviations

AF: angina frequency
AS: angina stability
CHD: coronary heart disease
CI: confidence interval
CNKI: China National Knowledge Infrastructure
CVD: cardiovascular disease
DAPT: dual antiplatelet therapy
DP: disease perception
HXHY: Huoxue Huayu therapy
ISR: in-stent restenosis
ITT: intention-to-treat analysis
MACE: major adverse cardiovascular events
MD: mean difference
MI: myocardial infarction
PCI: percutaneous coronary intervention
RR: relative risk
SAQ: Seattle Angina Questionnaires
TCM: traditional Chinese Medicine
TS: treatment satisfaction
